# Behavioural Problems in Developmental Disorders: A Paediatrician's Guide

**DOI:** 10.1111/jpc.70592

**Published:** 2026-09-30

**Authors:** Daryl Efron

**Affiliations:** ^1^ Royal Children's Hospital Melbourne Victoria Australia; ^2^ Murdoch Children's Research Institute Melbourne Victoria Australia; ^3^ Department of Paediatrics University of Melbourne Melbourne Victoria Australia

**Behavioural Problems in Developmental Disorders: A Paediatrician’s Guide. By Stewart Einfeld, Michael McDowell, and Adrian Sandler, Oxford: Oxford University Press, 2026, ISBN (online): 978‐0‐19‐198626‐0; ISBN (print): 978‐0‐19‐289967‐5**

This book addresses an important component of the modern paediatric caseload: behavioural problems in children and adolescents with developmental disorders.

The text is organised logically, progressing from causation and assessment through to formulation and management. The authors encourage behavioural formulation rather than diagnostic categorisation. Problematic behaviour is seen as the manifestation of interacting biological, developmental, psychological, environmental and social factors. This framework gives the book a rich coherent foundation that will resonate with clinicians who work with complex presentations.

Their use of the ‘5P’ framework—predisposing, precipitating, perpetuating, protective and palliating factors—encourages clinicians to develop nuanced and testable hypotheses about behaviour, rather than relying solely on diagnostic shorthand. The authors caution against reaching too quickly for the prescription pad.

Another major strength is the book's clinical practicality. The text is written by experienced physicians who understand the realities of busy clinical practice. Numerous case examples ground the discussion in real‐world paediatrics and illustrate how developmental level, communication ability, medical comorbidity, family stressors and educational environments all shape behavioural presentations. Practical tips (e.g., Unpredictable behaviour, Bullying) add to the book's clinical usefulness.

The authors also succeed in bridging disciplines that are too often siloed. The book sits comfortably at the interface of paediatrics, psychiatry, psychology and disability medicine, whereas remaining firmly anchored in paediatric clinical practice. The section addressing behavioural phenotypes associated with specific genetic syndromes is useful for clinicians seeking insights beyond diagnostic criteria.

This book does not attempt to provide detailed behavioural therapy or psychopharmacology guidance. Rather it describes a clinically useful conceptual framework for understanding behaviour problems in developmental disorders. The tone throughout is thoughtful and compassionate.

This excellent book will be of value for paediatricians, child psychiatrists, psychologists and allied health professionals working with children and adolescents with neurodevelopmental disorders.

## Funding

The author has nothing to report.

## Conflicts of Interest

The author declares no conflicts of interest.

## Data Availability

Data sharing not applicable to this article as no datasets were generated or analysed during the current study.

